# Associations between Periodontal Microbiota and Death Rates

**DOI:** 10.1038/srep35428

**Published:** 2016-10-17

**Authors:** Chung-Jung Chiu, Min-Lee Chang, Allen Taylor

**Affiliations:** 1 Jean Mayer United States Department of Agriculture Human Nutrition Research Center on Aging, Tufts University, Boston, MA, USA

## Abstract

It is conceived that specific combinations of periodontal bacteria are associated with risk for the various forms of periodontitis. We hypothesized that such specificity is also related to human cause-specific death rates. We tested this hypothesis in a representative sample of the US population followed for a mean duration of 11 years and found that two specific patterns of 21 serum antibodies against periodontal bacteria were significantly associated with increased all-cause and/or diabetes-related mortalities. These data suggested that specific combinations of periodontal bacteria, even without inducing clinically significant periodontitis, may have a significant impact on human cause-specific death rates. Our findings implied that increased disease and mortality risk could be transmittable via the transfer of oral microbiota, and that developing personalized strategies and maintaining healthy oral microbiota beyond protection against periodontitis would be important to manage the risk.

Mucosal surfaces, including the oral mucosa, are colonized by a complex and dynamic microbial ecosystem called “microbiota” that has important implications for human health and disease^1^,^2^. While more epidemiological evidence is warranted, periodontal microbiota has been identified as a causative agent of periodontitis, which is one of the most prevalent diseases in human population^3^,^4^. Interestingly, some animal and human observational evidence supports that periodontitis is not just an oral, *in situ* disease. The disease also contributes to several systemic diseases including diabetes and cardiovascular diseases (CVD)^5^,^6^,^7^,^8^,^9^. The chronic inflammatory processes of periodontitis are considered to be responsible for the etiologies^10^. In the oral cavity, the inflammatory and immunologic reactions following periodontitis induce the production of pro-inflammatory cytokines resulting in the breakdown of periodontal epithelium and connective tissues^2^,^11^. Systematically, the chronic trickling of periodontal microbiota into the systemic bloodstream elicits a systemic inflammation response resulting in elevated levels of various inflammatory mediators and cross-reactive systemic antibodies, which promote risk for many systemic diseases^12^,^13^,^14^,^15^. Importantly, it has been shown that the increased periodontitis-related all-cause and CVD mortalities are comparable with, but independent of, diabetes-related mortality^16^,^17^,^18^,^19^,^20^,^21^,^22^,^23^,^24^,^25^.

Results from a recent clinical trial, the Diabetes and Periodontal Therapy Trial (DPTT), do not support the use of nonsurgical periodontal treatment in patients with diabetes and periodontitis for the purpose of lowering levels of glycated hemoglobin (HbA1c)^26^. However, it is important to note that the trial did not evaluate the changes of periodontal microbiota after periodontal treatment. Studies have demonstrated that the re-development of oral microbiota was so rapid that the abundance of many pathogenic bacterial species exceeded the baseline values in two days after periodontal treatment^27^. It is likely that the failure to benefit from periodontal treatment was due to the failure to establish a commensal microbiota after the treatment.

It is believed that complex interactions between specific periodontal pathogens and different bacterial combinations are more relevant to periodontitis than are individual species^28^,^29^,^30^. We therefore hypothesize that a similar phenomenon exists in the association between periodontal microbiota and mortality rates. To test our hypothesis, we related 21 serum immunoglobulins G (IgGs) against periodontal bacteria to the rates of all-cause, diabetes-related, and hypertension-related mortalities in a death cohort from a representative sample of the US population, the Third National Health and Nutrition Examination Survey (NHANES III).

## Methods

### Study cohort

The NHANES III was performed between 1988 and 1994 by the National Center for Health Statistics (NCHS)^31^. It is a cross-sectional nationwide health survey of 33994 non-institutionalized US residents aged 2 months and older using a stratified multistage probability sampling design to sample a representative cohort of the US general population. The survey protocol was approved by the Institutional Review Board of the Centers for Disease Control and Prevention. All participants gave written informed consent. This study has been conducted according to the principles expressed in the Declaration of Helsinki.

All available sera from NHANES III (1988–1994) participants 40+ years old (n = 8153) were tested for the presence and level of 21 IgG antibodies against a broad panel of periodontal bacteria using a rapid checkerboard immunoblotting technique^32^,^33^. We excluded persons with missing IgG data, history of diabetes, heart attack, stroke, or cancers. Participants on immunomodulatory medications or corticosteroids were also excluded from our study. Among non-smokers, other tobacco product users such as chewing tobacco, cigar, and pipe and cotinine level >15 ng/ml were also excluded. We used the linked mortality-follow-up data through December 31, 2011 for these NHANES III participants and identified 1908 deaths (Table 1). The mean follow-up time to death was 10.9 years. A death was coded diabetes-related if ICD-9 code ‘250’ or ICD-10 codes ‘E10 thru E14’ was coded in the entity-axis multiple cause of death codes. For hypertension-related death, it was ICD-9 codes ‘401’ or ‘403’ or ICD-10 codes ‘I10’ or ‘I12’. Among this death cohort, 113 deaths were diabetes-related and 240 deaths were hypertension-related.

### Serum immunoglobulins G against periodontal bacteria

The following bacterial strains were used to prepare whole cell antigenic extracts for determining the levels of 21 IgG antibodies by means of the “checkerboard” immunoassay: *Aggregatibacter actinomycetemcomitans* ATCC strains #43718, #29523 and #33384; *Porphyromonas gingivalis* ATCC #33277 and #53978; *Tannerella forsythia* ATCC#43037 (TF); *Treponema denticola* OMGS#3271 (TD); *Campylobacter rectus* ATCC#33238 (CR); *Eubacterium nodatum* ATCC#33099 (EN); *Prevotella intermedia* ATCC#25611 (PI); *Prevotella nigrescens* ATCC#33563 (PN); *Prevotella melaninogenica* ATCC#25845 (PM); *Fusobacterium nucleatum* ATCC#10953 (FN); *Micromonas micros* ATCC #33270 (MM); *Selenomonas noxia* ATCC#43541 (SN); *Eikenella corrodens* ATCC#23834 (EC); *Capnocytophaga ochracea* ATCC#33624 (CO); *Streptococcus intermedius* ATCC#27335 (SI); *Streptococcus oralis* ATCC#35037 (SO); *Streptococcus mutans* ATCC#25175 (SM); *Veillonella parvula* ATCC#10790 (VP); and *Actinomyces naeslundii* ATCC#49340 (AN). To assess the level of antibody to *Porphyromonas gingivalis*, a mixed suspension of ATCC strains #33277 and #53978 (PGMX) was used. For the assessment of antibody to *Aggregatibacter actinomycetemcomitans*, a mixed suspension of three strains (ATCC#43718, #29523 and #33384; AAMX) was used as well as individual preparations from *Aggregatibacter actinomycetemcomitans* serotype a (ATCC strain #29523, AA29) and serotype b (ATCC strain #43718, AAY4).

**Figure 1:**
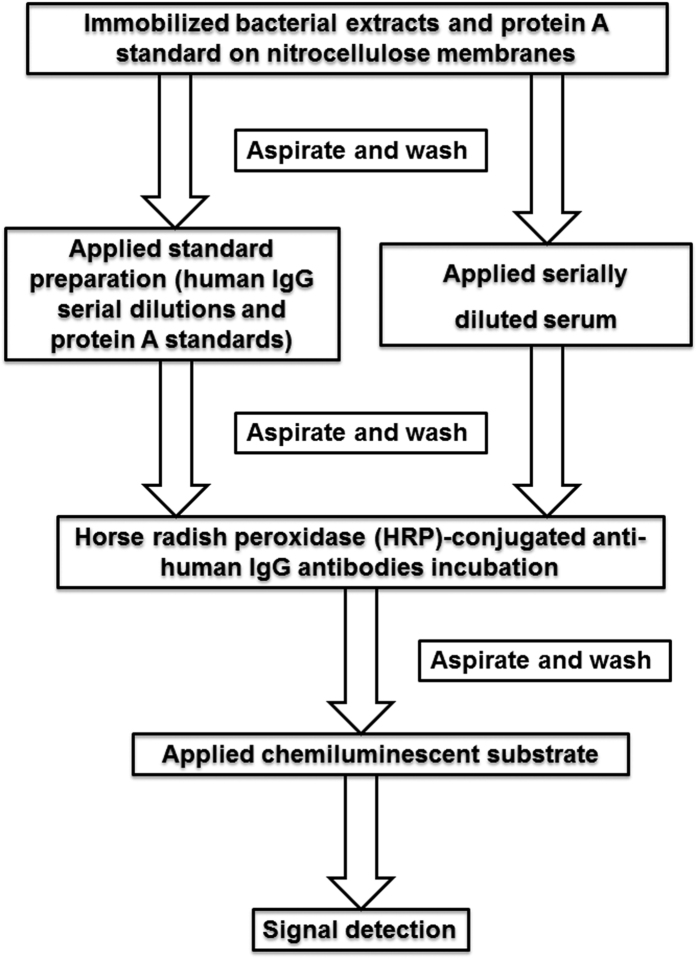
Flow diagram summarizes experimental procedures for checkerboard immunoassay. Whole cell bacterial antigenic extracts were used for determining the levels of IgG antibodies. The whole cell antigenic extracts and protein A standards were immobilized on nitrocellulose membranes. Serially diluted (1/250, 1/500 and 1/1000) serum from each subject as well as human IgG standards (250 ng/ml and 125 ng/ml) were loaded perpendicularly to the bacterial extracts, and were allowed to interact. After several washing steps, membranes were incubated with Fab fragments of anti-human IgG conjugated with horseradish-peroxidase and a horseradish-peroxidase substrate. The chemiluminescent signal was assessed in a LumiImager^TM^ Workstation. Signals were compared to the ones generated by the protein A and human IgG standards and expressed in a scale of 0 to 9. Whenever signal was present at several serum dilutions, the signal generated by the highest dilution was used to represent the particular patient’s antibody titer. IgG: immunoglobulin G.

Detailed laboratory procedures have been described elsewhere and summarized in Fig. 1 32. Briefly, each bacterial colony was cultivated on a Trypticase soy agar plate supplemented with 5% sheep blood. After 2 days of incubation period, colonies were harvested and suspended in phosphate buffered saline (PBS, pH 7.4), and sequentially sonicated on ice for 10 seconds with a micro-ultrasonic cell disrupter (Kontes, Vineland, NJ). The optical density of each suspension was adjusted to 1.0 at 600 nm, using a spectrophotometer (Ultrospec III, Parmacia, Sweden). The qualifications of the antigens have been validated with enzyme-linked immunosorbent assay (ELISA)^32^. The whole cell antigenic extracts derived from each of the above species and protein A standards were immobilized on nitrocellulose membranes. Serially diluted (1/250, 1/500 and 1/1000) serum from each subject as well as human IgG standards (250 ng/ml and 125 ng/ml) were loaded perpendicularly to the bacterial extracts, and were allowed to interact. After several washing steps, membranes were incubated with Fab fragments of anti-human IgG conjugated with horseradish-peroxidase and a horseradish-peroxidase substrate. The chemiluminescent signal was assessed in a LumiImager^TM^ Workstation. Signals were compared to the ones generated by the protein A and human IgG standards and expressed in a scale of 0 to 9. Whenever signal was present at several serum dilutions, the signal generated by the highest dilution was used to represent the particular patient’s antibody titer.

All assessed antibody titer levels are reported in the units of μg/ml in this data release. The mean (+/−standard error (SE)) of the coefficients of variation (CVs) for replicates within an assay averaged 7.3 (+/−2.3%)^32^. The CVs of the assay run on different days for serum antibody to a range of subgingival species averaged 10.1 (+/−2.1%) using the beaded protein A matrix preparation and 16.1 (+/−4.4%) using protein A solution directly on the membrane. Antibody present at levels >5 μg/ml were detectable in all replicates of each sample.

### Serum levels of vitamin C, vitamin E, lutein/zeazanthin, and C reactive protein

Details about the procedures and quality control systems for the determination of serum levels of vitamin C, vitamin E (alpha-tocopherol), lutein/zeazanthin, and C reactive protein (CRP) in the NHANES III have been described elsewhere^34^,^35^. In brief, vitamin C levels were measured by high-performance liquid chromatography (HPLC) with an electrochemical detector. Levels of vitamin E and lutein/zeazanthin were measured by using isocratic HPLC with detection at two different wavelengths. Serum CRP was measured with an automated Behring Nephelometer Analyzer System (Behring Diagnostics, Inc, Somerville, NJ).

### Assessment of periodontal health

Details of the oral health component of the NHANES III are published elsewhere^36^. The NHANES III periodontal measurements included periodontal probing depth (PPD), gingival recession and bleeding on probing (BOP). Clinical attachment loss (CAL) was calculated as the sum of the recession and PPD. To measure these periodontal measurements, the teeth in each participant were divided into two maxillary and two mandibular halves and measurements were taken from two sites per tooth (mid-buccal and mesio-buccal) for all teeth (excluding third molars) in one randomly chosen upper and lower quadrant. We employed continuous periodontal parameters for mean number of tooth sites that bled on probing (mBOP) and mean CAL (mCAL).

### Statistical methods

**Figure 2:**
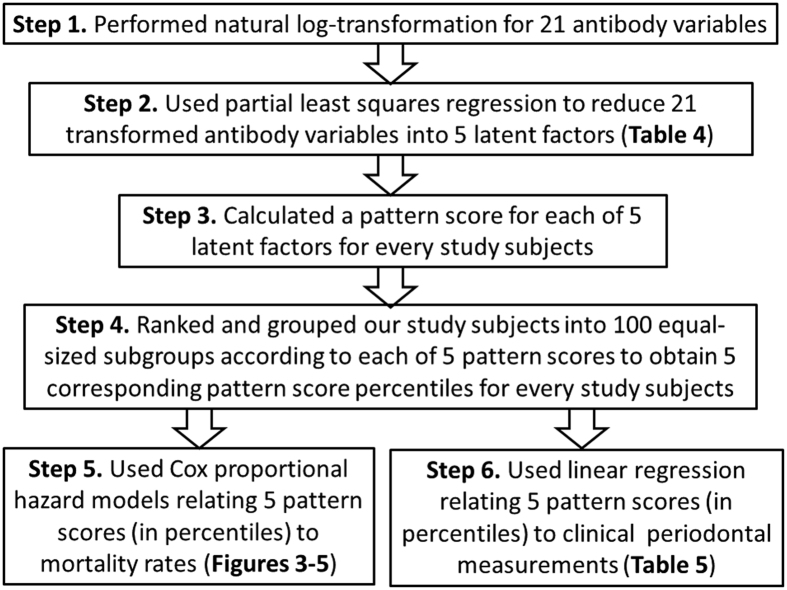
Statistical analysis consists of six steps. Step 1: Log-transformation. Step 2: Periodontal microbiota patterns by partial least squares regression. Step 3: Pattern (latent factor) score calculation. Step 4: Pattern score percentile ranking. Step 5: Periodontal microbiota patterns and mortalities association analysis. Step 6: Periodontal microbiota patterns and clinical periodontal measurements association analysis.

We summarized our analysis process in Fig. 2. First, because the distributions of the 21 IgG antibody levels showed positively skewed, before performing further analysis we first took a natural log for each of the IgG variables (Step 1 in Fig. 2). The log-transformed IgG variables were tested for normality and showed no significant deviations. These log(IgG) variables were used in further analysis.

We first explored the associations between 21 individual IgG variables and total, diabetes-related, and hypertension-related mortalities (Supplementary Figure 1a–c, respectively). Although we entered the 21 IgG variables into the model at the same time, due to the highly collinearity among the 21 IgG variables, residual confounding remained being an issue. Therefore, the results were not used in making our conclusion. Furthermore, we aimed to assess the combined effect of the 21 highly correlated IgG antibodies on mortality rates. To achieve this objective, we used partial least squares (PLS) regression to assemble the 21 highly collinear IgG variables into another 21 uncorrelated factors (latent factors) that describe maximum correlation between the 21 IgG variables and two mortality variables (diabetes-related and hypertension-related mortalities) (Step 2 in Fig. 2). Each PLS-derived latent factor can be considered as a specific periodontal microbiota pattern. According to the PLS analysis, a pattern score for each of the latent factors can be calculated for every study subjects. A pattern score was calculated by summarizing the 21 log(IgG) variables weighted by their corresponding loadings derived from the PLS analysis (Step 3 in Fig. 2). A higher loading indicates a more important contribution of the specific IgG to the pattern score. Therefore, a pattern score for each of the latent factors represented a specific combination of periodontal bacteria. Next, we ranked and grouped our study subjects into 100 equal-sized subgroups (i.e. percentiles) according to each of the pattern scores and assigned the percentiles as the values for latent factor score percentile variables (Step 4 in Fig. 2). Using Cox proportional hazard models, the latent factor score percentile variables were then individually related to cause-specific mortality rates (Step 5 in Fig. 2). To evaluate if periodontal measurements, mBOP and mCAL (see ***Assessment of periodontal health*** section), are related to the latent factors, we run a linear regression using either mBOP or mCAL as the dependent variable and each of the individual latent factor score percentile variables as the independent variable (Step 6 in Fig. 2).

The following were considered as covariates in our regression analysis: age, sex, race, education level, smoking status, body mass index (BMI, computed from weight and height; Kg/m^2^), drinking alcohol (at least 12 drinks in the past 12 months), and serum levels of CRP, vitamin C, vitamin E, and lutein/zeaxanthin. However, we also examined the results derived from models without including vitamin C, vitamin E, and lutein/zeaxanthin variables. The results were similar and led to the same conclusion with the results from models including the three variables.

Our analyses were performed using SAS^®^ *SURVEY* procedures (version 9.3; SAS Institute Inc, Cary, NC), which take into account of the complex sampling design used in NHANES III and yield unbiased SE and confidence interval (CI) estimates. We used *P* < 0.05 to denote statistical significance and all tests were two-sided.

## Results

### Characteristics of death cohorts

Table 1 shows the baseline characteristics of our study death cohort consisting of 1908 deaths, including 113 diabetes-related deaths and 240 hypertension-related deaths, after 10.9 years of follow-up in the NHANES III. Compared with non-diabetes-related deaths, diabetes-related deaths had higher BMI and lower serum levels of lutein/zeaxanthin (Table 2). There were marginally significant (0.05 < P < 0.1) differences in age, sex, race, and serum levels of CRP between the two groups. In terms of hypertension-related deaths (Table 3), they had significantly higher BMIs and tended to be non-Hispanic black than non-hypertension-related deaths. They also tended to be females but did not reach the significant level.

### Partial least squares regression

As described in the ***Statistical methods*** section, using the PLS technique we reduced the 21 highly collinear IgG variables to a smaller set of uncorrelated latent factors. We decided to focus on the top five latent factors (Factors 1–5) derived from our PLS analysis because they met our preset criterion of accounting for over 70% of total variation in the 21 IgG variables. It is to say that we used PLS regression to reduce the 21 highly collinear IgG variables into five uncorrelated factors (latent factors) that described maximum correlation between independent (the 21 IgG) variables and dependent (mortality) variables. Factor 1 accounted for the largest variation (0.79%) in the two mortality variables (diabetes-related and hypertension-related mortalities) but only for a small variation (5.40%) in the 21 IgG variables. Factor 3 accounted for the largest variation (38.30%) in the 21 IgG variables followed by Factor 2 (17.89%), and they accounted for 0.13% and 0.29% respectively in the two mortality variables. Factor 4 and Factor 5 accounted for only small amounts of variations both in the 21 IgG variables (5.12% and 3.37% respectively) and in the two mortality variables (0.24% and 0.16% respectively).

Table 4 shows the PLS model effect loadings of the 21 IgG variables in each latent factor (Factor). A higher loading indicates a more important contribution of the specific IgG to the Factor, which represents a specific combination of periodontal microbiota. Accordingly, a pattern score for each of the Factors can be calculated for every study subjects by summarizing the 21 log(IgG) variables weighted by their corresponding loadings.

**Figure 3:**
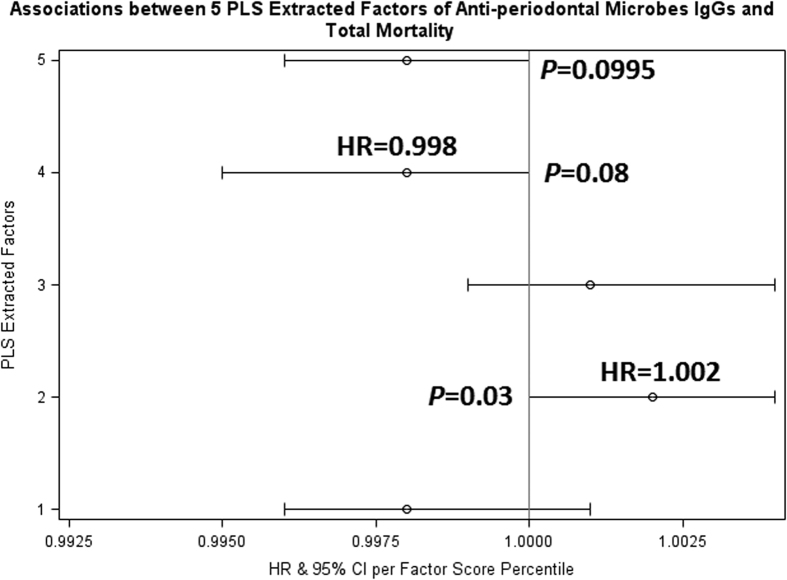
Cox proportional hazard regression analysis relating five partial least squares latent factors to all-cause death rate. Models were adjusted for age, sex, race, education level, smoking status, body mass index, drinking alcohol (at least 12 drinks in the past 12 months), and serum levels of C reactive protein, vitamin C, vitamin E and lutein/zeaxanthin, and the sampling weights in the Third National Health and Nutrition Examination Survey. IgG: immunoglobulin G. PLS: partial least squares. HR: hazard ratio. CI: confidence interval.

**Figure 4:**
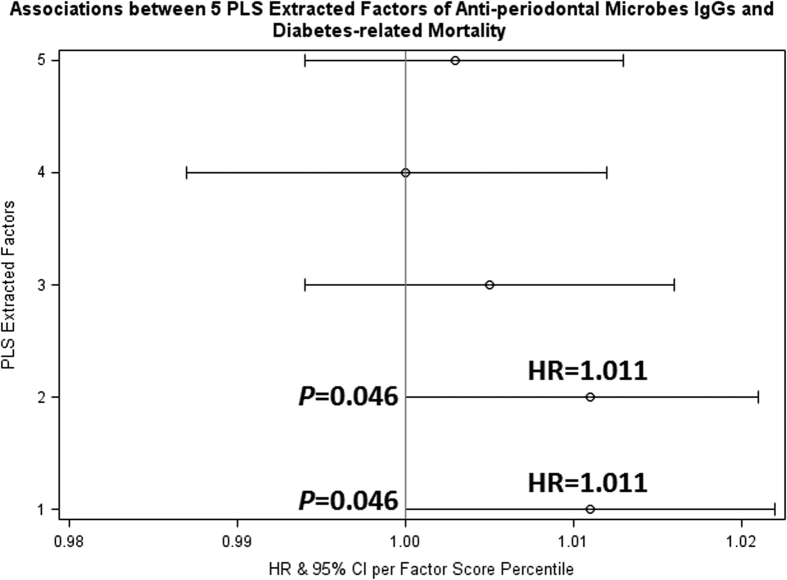
Cox proportional hazard regression analysis relating five partial least squares latent factors to diabetes-related death rate. Models were adjusted for age, sex, race, education level, smoking status, body mass index, drinking alcohol (at least 12 drinks in the past 12 months), and serum levels of C reactive protein, vitamin C, vitamin E and lutein/zeaxanthin, and the sampling weights in the Third National Health and Nutrition Examination Survey. IgG: immunoglobulin G. PLS: partial least squares. HR: hazard ratio. CI: confidence interval.

Factor 1 featured heavy loadings (≥0.2) in the IgGs against PGMX, PI, PN, and the three AA strains, but lower loadings (≤−0.2) in the IgGs against TF, EC, EN, VP, SN, and TD. Interestingly, PG, PI, PN, and the three AA strains have been shown to be strongly associated with periodontitis and our analysis also indicated that Factor 1 suggests active periodontitis (see Table 5). Factor 2 featured low loadings in all IgGs except the IgG against PGMX although the loading for PGMX was small (0.02); in other words, it featured a relatively high loading only in the IgG against PGMX. This data is consistent with that PG can serve as a keystone pathogen in periodontitis. The two latent factors were significantly associated with all-cause and/or diabetes-related mortalities (see Figs 3 and 4).

Factor 3 featured high loadings in all IgGs, especially (≥0.2) the IgG against PGMX, TF, the three AA strains, SO, CR, EC, EN, SI, VP, SN, TD, and SM. Factor 3 can be considered as an enhanced host immune responses resulting in high levels in all of the 21 IgGs.

Factor 4 featured low loadings in most IgGs except the IgGs against SO, MM, EN, SI, AN, and SM. Factor 5 were heavily loaded (≥0.2) in IgGs against PN, CO, PM, and SM, but featured lower loadings (≤−0.2) in IgGs against the three AA strains, CR, EC, EN, and AN. In contrast to Factor 1, Factor 4 featured low loadings in the IgGs against PGMX, PI, PN and the three AA strains and Factor 5 featured low loadings in the IgGs against PGMX, PI, and the three AA strains. Therefore, Factor 4 and Factor 5 can be considered as two reflections of Factor 1 in terms of the loadings for the characteristic bacterial antibodies.

### Serum immunoglobulin G patterns and clinical periodontal measurements

As defined in the ***Assessment of periodontal health*** section, BOP can be considered as a sign of periodontal inflammation and CAL can be considered as a measure of the extent of the periodontal support that has been destroyed around a tooth. We used mean BOP (mBOP) as a surrogate to the activity of periodontitis and mean CAL (mCAL) as a surrogate to the overall periodontal destruction (severity) for a subject. Table 5 shows the relationships between the two periodontal measurements and the five latent factors. Accordingly, we described Factor 1 as an indicator of “active periodontitis”, Factor 2 as an indicator of “no clinically significant periodontitis”, Factor 3 as an indicator of “inactive periodontitis”, Factor 4 as an indicator of “inactively protective against periodontitis”, and Factor 5 as an indicator of “actively protective against periodontitis”.

### Serum immunoglobulin G patterns and mortalities

**Figure 5:**
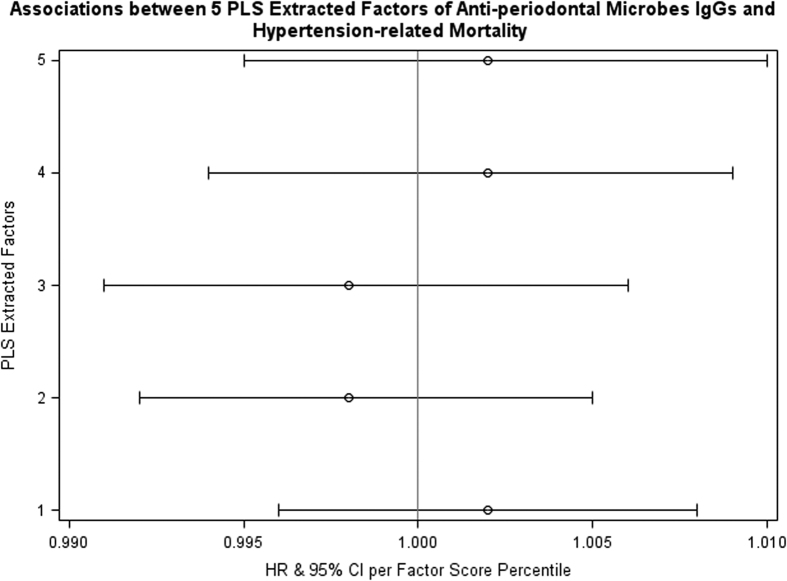
Cox proportional hazard regression analysis relating five partial least squares latent factors to hypertension-related death rate. Models were adjusted for age, sex, race, education level, smoking status, body mass index, drinking alcohol (at least 12 drinks in the past 12 months), and serum levels of C reactive protein, vitamin C, vitamin E and lutein/zeaxanthin, and the sampling weights in the Third National Health and Nutrition Examination Survey. IgG: immunoglobulin G. PLS: partial least squares. HR: hazard ratio. CI: confidence interval.

In our Cox regression analysis, we found that, per percentile increased in Factor 2 score, there was a 0.2% increase in total mortality rate (hazard ratio (HR)  = 1.002; 95% CI: 1.000 to 1.004); in other words, compared with people in the lowest percentile of Factor 2 score, those in the highest percentile of Factor 2 score had 20% higher all-cause mortality rate (Fig. 3). Further adjustment for the status of diabetes-related mortality did not change the results. There were marginally significantly (*P* = 0.08 and *P* = 0.0995, respectively) lower all-cause mortality rate per percentile increased in Factor 4 score and Factor 5 score (HRs = 0.998). In our diabetes-related mortality analysis (Fig. 4), we found that higher scores in either Factor 1 or Factor 2 were significantly associated with high rates of death due to diabetes-related causes; per percentile increased in the scores was related to a 1.1% increase in diabetes-related death. In other words, compared with people in the lowest percentile of Factor 1 or Factor 2 score, those in the highest percentile of Factor 1 or Factor 2 score had over one-fold higher rate of death due to diabetes-related causes. No significant relationship was noted between hypertension-related mortality and any of the five pattern scores (Fig. 5).

## Discussion

In this study, we found that two baseline serum IgG patterns, Factor 1 and Factor 2, were significantly associated with higher all-cause and/or diabetes-related mortality rates among people without history of diabetes, CVD, and cancers. While only Factor 2 was related to all-cause mortality, both Factor 1 and Factor 2 were related to diabetes-related mortality. To our best knowledge, this is the first data showing that specific oral microbiota may have an impact on the rate of death in humans.

Different from most studies in human microbiota, which measure bacterial DNA to evaluate the quantities and species of microbiome, we used serum IgGs against periodontal bacteria in this study. Serum IgGs reflected human systemic response to the corresponding periodontal bacteria and studies have shown that individual periodontal bacterial quantities were significantly correlated with corresponding serum antibody levels^37^. Therefore, the serum IgG levels can be considered as host-related phenotypes of periodontal microbiota.

Our analysis showed that, although the two mortality-related IgG patterns that we characterized featured several bacteria, which were also featured in periodontitis-related complexes, they were in different combinations. It seemed that different bacterial combinations have different impacts on human health. For example, Socransky et al. identified five microbial complexes, which were repeatedly found together in subgingival biofilm^28^. Among these, the “red complex”, consisting of PG, TF, and TD, is considered the most pathogenic microbial complex for periodontitis^38^. Our Factor 1 (an indicator of active periodontitis; see Table 5) featured heavy loadings in the IgGs against PGMX, PI, PN, and the three AA strains, all of which were strongly associated with periodontitis^28^. However, unlike Socransky’s red complex, our Factor 1 featured low loadings in the IgGs against TF and TD. Similarly, our Factor 2 (an indicator of no clinically significant periodontitis) featured a relatively high loading (with a small value of loading factor of 0.02, see Table 4) only in the IgG against PGMX but low loadings in all the other IgGs, including the two IgGs against TF and TD. Interestingly, our findings coincide with the hypothesis of PG as a keystone pathogen. It is conceived that the mere presence of a keystone pathogen, even at very low colonization levels, can modulate host response in ways that alter the amount and composition of subgingival microbiota, thereby triggering adverse effects on human health^29^,^39^. It has been demonstrated in a murine periodontal model that the introduction of PG, even at low numbers, in cooperation with other dysbiotic bacteria led to a marked acceleration in pathological alveolar bone loss, but PG alone failed to induce periodontitis^39^. Importantly, our findings from Factor 1 and Factor 2 also, respectively, suggested that active periodontitis may increase diabetes-related death rate, and that, even without clinically significant periodontitis, the presence of PG at very low colonization levels increase total and diabetes-related death rate. It seemed that the elimination of PG is crucial in reducing risk for both periodontitis and mortality.

Despite of featuring high loadings in all of the 21 IgGs, we did not find an association between Factor 3 and mortalities. As Factor 3 indicating an inactive periodontitis, it was likely that Factor 3 reflected an enhanced host immune responses resulting in a periodontal bacterial consortium which was constrained to a homeostasis. It was also noteworthy that, in contrast with Factor 1, which featured heavy loadings in the IgGs against PGMX, PI, PN and the three AA strains and showed a significant association with diabetes-related mortality but no association with total mortality, Factor 4 (featuring low loadings in the IgGs against PGMX, PI, PN and the three AA strains) and Factor 5 (featuring low loadings in the IgGs against PGMX, PI, and the three AA strains) suggested protective against total mortality but no association with diabetes-related mortality. Our data provided self-consistent evidence that the suppression of this specific bacterial combination, PGMX, PI, and the three AA strains, may be beneficial. However, studies have suggested that a specific combination of periodontal bacterial species may have important implication on a human disease only in a particular population^29^. Our findings in a representative US cohort may be different from those in other populations.

Our findings collaborated with previous observations that periodontitis, a result of polymicrobial infection, increased the risk for several major diseases, such as diabetes, CVD, cancers^40^,^41^,^42^,^43^, and mortalities as well^16^,^17^,^18^,^19^,^20^,^21^,^22^,^23^,^24^,^25^. The etiologies may involve several pathological consequences leading to uncontrolled inflammation, such as elevated levels of systemic proinflammatory cytokines^14^,^44^,^45^, oxidative stress^46^,^47^, formation of advanced glycation end products^48^, disturbed microbe-host nutrition and metabolism interaction^49^,^50^,^51^, etc. These mechanisms may be responsible not only for the initiation but also for the promotion and progression of the diseases as well, and thus lead to higher death rates. However, it has been shown that periodontal microbial interactions are complex and that numerous genes related to motility, metabolism, and virulence in one bacterium are differentially regulated in the presence of others^29^,^52^,^53^. The detailed mechanisms relating specific combinations of periodontal bacteria to specific diseases or death rates warrant further study. The information would be valuable in developing personalized therapeutic and prevention strategies.

The strengths of this study included a prospective study in a representative cohort of the US population, standardized collection of risk factor information and periodontal examinations to minimize the influence of confounding factors and misclassifications. However, the ascertainment of cause-specific death in the NHANES III may not comprehensive enough, especially for the hypertension-related death used in this study. The limited number of diabetes-related deaths in our study resulted in inadequate sample sizes for some analyses. Among the 1908 apparently healthy study subjects, 1792 (93.9%) had missing information for taking cholesterol lowing medications. This hindered us in addressing the potential confounding issue, especially for our analysis on the hypertension-related mortality. Although serum IgGs are considered to reflect chronic, intermittent exposure^54^, the one-time measurement at baseline may not reflect a long-term exposure to dynamic periodontal microbiota.

In conclusion, our analysis suggested that specific combinations of periodontal bacteria, even without inducing clinically significant periodontitis, may have a significant impact on human cause-specific death rates. More mechanistic and human observational studies are needed before a clinical trial could be implemented to confirm our findings. If an etiological relationship of specific periodontal microbiota to death rates is established, increased mortality could be transmittable via the transfer of oral microbiota. In that case, developing personalized strategies and maintaining a healthy oral microbiota, beyond that against periodontitis, would be important to manage the increased mortality risk.

## Additional Information

**How to cite this article**: Chiu, C.-J. *et al*. Associations between Periodontal Microbiota and Death Rates. *Sci. Rep.* **6**, 35428; doi: 10.1038/srep35428 (2016).

## Supplementary Material

Supplementary Information

## Figures and Tables

**Table 1:** Baseline characteristics of death cohort consisting of 1908 deaths after 10.9 years of follow-up in the Third National Health and Nutrition Examination Survey (NHANES III).

Baseline Characteristics	Total Death (n = 1908)
**Age at interview**	65.43 (0.56)
**Sex**	
*Female*	963 (55.32%)
*Male*	945 (44.68%)
**Race**	
*Non-Hispanic white*	1072 (85.90%)
*Non-Hispanic black*	476 (11.10%)
*Mexican-American*	360 (2.99%)
**Education Years**	
<*12 yrs*	1018 (37.96%)
*12 yrs*	492 (33.54%)
>*12 yrs*	398 (28.51%)
**Smoking Status**	
*Never smoker*	736 (35.83%)
*Past smoker*	628 (33.64%)
*Current smoker*	544 (30.53%)
**Alcoholic Drinking**	
*No*	805 (52.36%)
*Yes*	662 (47.64%)
**Body mass index (Kg/m**^**2**^)	27.16 (0.18)
**Serum levels**	
*Vitamin C (mmol/L)*	46.53 (1.35)
*Vitamin E (umol/L)*	31.24 (0.48)
*Lutein/Zeazanthin (umol/L)*	0.40 (0.0095)
*C-reactive Protein (mg/dL)*	0.58 (0.028)

Values which are not followed by percentages are means (standard error). All values, except values (sample sizes) followed by percentages, are weighted by the NHANES III sampling scheme.

**Table 2:** Baseline characteristics of 113 diabetes-related deaths after 10.9 years of follow-up in the Third National Health and Nutrition Examination Survey (NHANES III).

Baseline Characteristics	Diabetes-related Death		
	No (n = 1795)	Yes (n = 113)	*P*
**Age at interview**	65.60 (0.53)	62.54 (1.77)	0.058
**Sex**			
*Female*	903 (93.37%)	60 (6.63%)	0.094
*Male*	892 (96.11%)	53 (3.89%)	
**Race**			
*Non-Hispanic white*	1019 (94.68%)	53 (5.32%)	0.064
*Non-Hispanic black*	452 (94.65%)	24 (5.35%)	
*Mexican-American*	324 (91.96%)	36 (8.04%)	
**Education Years**			
*<12 yrs*	958 (95.06%)	60 (4.94%)	0.166
*12 yrs*	455 (92.52%)	37 (7.48%)	
*>12 yrs*	382 (96.42%)	16 (3.58%)	
**Smoking Status**			
*Never smoker*	697 (94.77%)	39 (5.23%)	0.269
*Past smoker*	596 (96.09%)	32 (3.91%)	
*Current smoker*	502 (92.74%)	42 (7.26%)	
**Alcoholic Drinking**			
*No*	756 (93.71%)	49 (6.29%)	0.205
*Yes*	626 (95.83%)	36 (4.17%)	
**Body mass index (Kg/m**^**2**^)	26.97 (0.19)	30.35 (1.05)	0.003
**Serum levels**			
*Vitamin C (mmol/L)*	46.82 (1.40)	41.32 (4.14)	0.207
*Vitamin E (umol/L)*	31.17 (0.47)	32.58 (2.75)	0.608
*Lutein/Zeazanthin (umol/L)*	0.40 (0.0098)	0.34 (0.0177)	0.003
*C-reactive Protein (mg/dL)*	0.56 (0.0304)	0.93 (0.1961)	0.081

Values which are not followed by percentages are means (standard error). All values, except values (sample sizes) followed by percentages, are weighted by the NHANES III sampling scheme and all *P* values comparing those who died from cause-specific deaths with those who didn’t have been adjusted for the sampling weights.

**Table 3:** Baseline characteristics of 240 hypertension-related deaths after 10.9 years of follow-up in the Third National Health and Nutrition Examination Survey (NHANES III).

Baseline Characteristics	Hypertension-related Death		
	No (n = 1668)	Yes (n = 240)	*P*
**Age at interview**	65.32 (0.60)	66.33 (1.13)	0.406
**Sex**			
*Female*	826 (87.11%)	137 (12.89%)	0.065
*Male*	842 (91.11%)	103 (8.89%)	
**Race**			
*Non-Hispanic white*	954 (89.39%)	118 (10.61%)	0.020
*Non-Hispanic black*	400 (84.61%)	76 (15.39%)	
*Mexican-American*	314 (90.50%)	46 (9.50%)	
**Education Years**			
*<12 yrs*	902 (90.84%)	116 (9.16%)	0.311
*12 yrs*	423 (87.00%)	69 (13.00%)	
*>12 yrs*	343 (88.54%)	55 (11.46%)	
**Smoking Status**			
*Never smoker*	635 (87.30%)	101 (12.70%)	0.536
*Past smoker*	556 (90.10%)	72 (9.90%)	
*Current smoker*	477 (89.44%)	67 (10.56%)	
**Alcoholic Drinking**			
*No*	699 (88.74%)	106 (11.26%)	0.666
*Yes*	585 (89.77%)	77 (10.23%)	
**Body mass index (Kg/m**^**2**^)	26.99 (0.19%)	28.48 (0.52)	0.008
**Serum levels**			
*Vitamin C (mmol/L)*	46.53 (1.30)	46.54 (3.47)	0.999
*Vitamin E (umol/L)*	31.24 (0.53)	31.24 (1.02)	0.993
*Lutein/Zeazanthin (umol/L)*	0.40 (0.01)	0.40 (0.0144)	0.724
*C-reactive Protein (mg/dL)*	0.58 (0.0304)	0.59 (0.0719)	0.866

Values which are not followed by percentages are means (standard error). All values, except values (sample sizes) followed by percentages, are weighted by the NHANES III sampling scheme and all *P* values comparing those who died from cause-specific deaths with those who didn’t have been adjusted for the sampling weights.

**Table 4:** Effect loadings of 21 serum periodontal bacterial immunoglobulins G for each of the five top latent variables derived from partial least squares model.

Latent factor	PGMX	PI	PN	TF	AAMX	AA29	AAY4	FN	SO	MM	CR	EC	EN	SI	CO	VP	AN	PM	SN	TD	SM
**Factor 1**	0.21	0.34	0.30	−0.20	0.25	0.25	0.32	−0.06	−0.09	−0.09	−0.09	−0.24	−0.21	0.05	−0.08	−0.25	−0.16	0.13	−0.25	−0.41	−0.04
**Factor 2**	0.02	−0.22	−0.23	−0.15	−0.20	−0.21	−0.22	−0.34	−0.27	−0.33	−0.23	−0.19	−0.02	−0.27	−0.24	−0.24	−0.08	−0.25	−0.20	−0.03	−0.26
**Factor 3**	0.24	0.17	0.19	0.27	0.26	0.25	0.23	0.18	0.25	0.15	0.22	0.20	0.23	0.21	0.19	0.23	0.16	0.16	0.24	0.26	0.24
**Factor 4**	−0.21	−0.42	−0.34	−0.26	−0.08	−0.12	−0.14	−0.05	0.07	0.006	−0.19	−0.29	0.19	0.05	−0.18	−0.18	0.26	−0.46	−0.16	−0.08	0.12
**Factor 5**	−0.16	−0.006	0.22	−0.08	−0.20	−0.23	−0.30	0.01	0.13	0.13	−0.36	−0.34	−0.35	0.07	0.27	0.10	−0.34	0.30	0.05	0.05	0.22

Partial least squares (PLS) regression was used to assemble the 21 highly collinear IgG variables into another 21 uncorrelated factors (latent factors) that describes maximum correlation between the 21 IgG variables and two mortality variables (diabetes-related and hypertension-related mortalities). However, only the top five latent factors (Factors 1–5) derived from the PLS analysis was retained because they met our preset criterion of accounting for over 70% of total variation in the 21 IgG variables. A higher effect loading indicates a more important contribution of the specific IgG to the Factor, which represents a specific combination of periodontal microbiota.

PLS: partial least squares.

PGMX: *Porphyromonas gingivalis*, a mixed suspension of ATCC strains #33277 and #53978.

PI: *Prevotella intermedia* ATCC#25611.

PN: *Prevotella nigrescens* ATCC#33563.

TF: *Tannerella forsythia* ATCC#43037.

AAMX: *Aggregatibacter actinomycetemcomitans*, a mixed suspension of three strains (ATCC#43718, #29523 and #33384).

AA29: *Aggregatibacter actinomycetemcomitans* serotype a (ATCC strain #29523).

AAY4: *Aggregatibacter actinomycetemcomitans* serotype b (ATCC strain #43718).

FN: *Fusobacterium nucleatum* ATCC#10953.

SO: *Streptococcus oralis* ATCC#35037.

MM: *Micromonas micros* ATCC #33270.

CR: *Campylobacter rectus* ATCC#33238.

EC: *Eikenella corrodens* ATCC#23834.

EN: *Eubacterium nodatum* ATCC#33099.

SI: *Streptococcus intermedius* ATCC#27335.

CO: *Capnocytophaga ochracea* ATCC#33624.

VP: *Veillonella parvula* ATCC#10790.

AN: *Actinomyces naeslundii* ATCC#49340.

PM: *Prevotella melaninogenica* ATCC#25845.

SN: *Selenomonas noxia* ATCC#43541.

TD: *Treponema denticola* OMGS#3271.

SM: *Streptococcus mutans* ATCC#25175.

**Table 5:** Linear regression coefficients and significance *P* values for each of the top five partial least squares latent variables vs. periodontitis activity and severity measured by mBOP and mCAL, respectively.

Latent factor	mBOP		mCAL		Clinical implication
	Coefficient	*P*	Coefficient	*P*	
**Factor 1**	0.00018	0.036	0.00285	<0.0001	Active periodontitis
**Factor 2**	0.00009	0.273	0.001	0.137	No clinically significant periodontitis
**Factor 3**	0.00011	0.166	0.00365	<0.0001	Inactive periodontitis
**Factor 4**	−0.0001	0.243	−0.002	0.0010	Inactively protective against periodontitis
**Factor 5**	−0.00022	0.008	−0.00322	<0.0001	Actively protective against periodontitis

The linear model used used either mBOP or mCAL as the dependent variable and each of the individual latent factor score percentile variables as the independent variable.

Mean number of tooth sites that bled on probing (mBOP) was used as an indicator of periodontitis activity. A significant (P<0.05) positive coefficient suggests that the higher the latent factor score, the more active the periodontitis. A significant negative coefficient suggests that the higher the latent factor score, the less active (i.e. more protective against) the periodontitis.

Mean clinical attachment loss (mCAL) was used as an indicator of periodontitis severity. A significant positive coefficient suggests that the higher the latent factor score, the more severe the periodontitis. A significant negative coefficient suggests that the higher the latent factor score, the less severe the periodontitis.

The clinical implication was derived from combining the information from mBOP and mCAL.

Models were adjusted for age, sex, race, education level, smoking status, body mass index, drinking alcohol (at least 12 drinks in the past 12 months), and serum levels of C reactive protein, vitamin C, vitamin E and lutein/zeaxanthin, and the sampling weights in the Third National Health and Nutrition Examination Survey.

## References

[b1] Honda K. Porphyromonas gingivalis sinks teeth into the oral microbiota and periodontal disease. Cell Host Microbe. 10, 423–425 (2011). 22100158 10.1016/j.chom.2011.10.008

[b2] Darveau R. P. Periodontitis: a polymicrobial disruption of host homeostasis. Nat Rev Microbiol. 8, 481–490 (2010). 20514045 10.1038/nrmicro2337

[b3] Burt B. & Research Science and Therapy Committee of the American Academy of Periodontology. Position paper: epidemiology of periodontal diseases. J Periodontol. 76, 1406–1419 (2005). 16101377 10.1902/jop.2005.76.8.1406

[b4] Chapple I. L. Time to take periodontitis seriously. BMJ. 348, g2645 (2014). 24721751 10.1136/bmj.g2645

[b5] Rosenstein E. D., Greenwald R. A., Kushner L. J. & Weissmann G. Hypothesis: the humoral immune response to oral bacteria provides a stimulus for the development of rheumatoid arthritis. Inflammation. 28, 311–318 (2004). 16245073 10.1007/s10753-004-6641-z

[b6] Kozarov E. V., Dorn B. R., Shelburne C. E., Dunn W. A. J. & Progulske-Fox A. Human atherosclerotic plaque contains viable invasive Actinobacillus actinomycetemcomitans and Porphyromonas gingivalis. Arterioscler Thromb Vasc Biol. 25, e17–18 (2005). 15662025 10.1161/01.ATV.0000155018.67835.1a

[b7] Genco R., Offenbacher S. & Beck J. Periodontal disease and cardiovascular disease: epidemiology and possible mechanisms. J Am Dent Assoc. 133, 14S-22S (2002). 10.14219/jada.archive.2002.0375 12085720

[b8] Chapple I. L., Genco R. & Working group 2 of the joint EFP/AAP workshop. Diabetes and periodontal diseases: consensus report of the Joint EFP/AAP Workshop on Periodontitis and Systemic Diseases. J Periodontol. 84, S106–112 (2013). 23631572 10.1902/jop.2013.1340011

[b9] Demmer R. T. . Periodontal Bacteria and Prediabetes Prevalence in ORIGINS: The Oral Infections, Glucose Intolerance, and Insulin Resistance Study. J Dent Res. 94, 201S–211S (2015). 26082387 10.1177/0022034515590369 PMC4547206

[b10] Southerland J. H., Taylor G. W., Moss K., Beck J. D. & Offenbacher S. Commonality in chronic inflammatory diseases: Periodontitis, diabetes, and coronary artery disease. Periodontol 2000 40, 130–143 (2006). 16398690 10.1111/j.1600-0757.2005.00138.x

[b11] Birkedal-Hansen H. Role of cytokines and inflammatory mediators in tissue destruction. J Periodontal Res 28, 500–510 (1993). 8263720 10.1111/j.1600-0765.1993.tb02113.x

[b12] Schenkein H. A. & Loos B. G. Inflammatory mechanisms linking periodontal diseases to cardiovascular diseases. J Clin Periodontol 40, S51–S69 (2013). 23627334 10.1111/jcpe.12060 PMC4554326

[b13] Choi H. M., Han K., Park Y. G. & Park J. B. Associations Among Oral Hygiene Behavior and Hypertension Prevalence and Control: The 2008 to 2010 Korea National Health and Nutrition Examination Survey. J Periodontol 86, 866–873 (2015). 25741579 10.1902/jop.2015.150025

[b14] Lalla E. & Papapanou P. N. Diabetes mellitus and periodontitis: a tale of two common interrelated diseases. Nat Rev Endocrinol. 7, 738–748 (2011). 21709707 10.1038/nrendo.2011.106

[b15] Noble J. M. . Serum IgG antibody levels to periodontal microbiota are associated with incident Alzheimer disease. PLoS One. 9, e114959 (2014). 25522313 10.1371/journal.pone.0114959 PMC4270775

[b16] Sharma P., Dietrich T., Ferro C. J., Cockwell P. & Chapple I. L. Association between periodontitis and mortality in stages 3-5 chronic kidney disease: NHANES III and linked mortality study. J Clin Periodontol. 43, 104–113 (2016). 26717883 10.1111/jcpe.12502 PMC5324563

[b17] Saremi A. . Periodontal disease and mortality in type 2 diabetes. Diabetes Care. 28, 27–32 (2005). 15616229 10.2337/diacare.28.1.27

[b18] Albert D. A. . Diabetes and oral disease: implications for health professionals. Ann N Y Acad Sci. May, 1255, 1251–1215 (2012). 10.1111/j.1749-6632.2011.06460.x PMC3429365 22409777

[b19] Li Q. . Oral disease and subsequent cardiovascular disease in people with type 2 diabetes: a prospective cohort study based on the Action in Diabetes and Vascular Disease: Preterax and Diamicron Modified-Release Controlled Evaluation (ADVANCE) trial. Diabetologia. 53, 2320–2327 (2010). 20700576 10.1007/s00125-010-1862-1 PMC4170775

[b20] Kshirsagar A. V. . Periodontal disease adversely affects the survival of patients with end-stage renal disease. Kidney International 75, 746–751 (2009). 19165177 10.1038/ki.2008.660

[b21] Chen L. P. . Relationship between periodontal disease and mortality in patients treated with maintenance hemodialysis. Am J Kidney Dis. 57, 276–282 (2011). 21177012 10.1053/j.ajkd.2010.09.016

[b22] de Souza C. M. . Association among oral health parameters, periodontitis, and its treatment and mortality in patients undergoing hemodialysis. J Periodontol 85, E169–E178 (2014). 24224959 10.1902/jop.2013.130427

[b23] Garcia R. I., Krall E. A. & Vokonas P. S. Periodontal disease and mortality from all causes in the VA Dental Longitudinal Study. Ann Periodontol 3, 339–349 (1998). 9722718 10.1902/annals.1998.3.1.339

[b24] Xu F. & Lu B. Prospective association of periodontal disease with cardiovascular and all-cause mortality: NHANES III follow-up study. Atherosclerosis 218, 536–542 (2011). 21831372 10.1016/j.atherosclerosis.2011.07.091

[b25] Linden G. J. . All-cause mortality and periodontitis in 60–70-year-old men: a prospective cohort study. J Clin Periodontol 39, 940–946 (2012). 22834905 10.1111/j.1600-051X.2012.01923.x

[b26] Engebretson S. P. . The effect of nonsurgical periodontal therapy on hemoglobin A1c levels in persons with type 2 diabetes and chronic periodontitis: a randomized clinical trial. JAMA. 310, 2523–2532 (2013). 24346989 10.1001/jama.2013.282431 PMC4089989

[b27] Uzel N. G. . Microbial shifts during dental biofilm re-development in the absence of oral hygiene in periodontal health and disease. J Clin Periodontol. 38, 612–620 (2011). 21488936 10.1111/j.1600-051X.2011.01730.x PMC3177321

[b28] Socransky S. S., Haffajee A. D., Cugini M. A., Smith C. & Kent R. L. J. Microbial complexes in subgingival plaque. J Clin Periodontol 25, 134–144 (1998). 9495612 10.1111/j.1600-051x.1998.tb02419.x

[b29] Torrungruang K., Jitpakdeebordin S., Charatkulangkun O. & Gleebbua Y. Porphyromonas gingivalis, Aggregatibacter actinomycetemcomitans, and Treponema denticola/Prevotella intermedia Co-Infection Are Associated with Severe Periodontitis in a Thai Population. PLoS One. 10, e0136646 (2015). 26313005 10.1371/journal.pone.0136646 PMC4552424

[b30] Lourenço T. G. . Microbial signature profiles of periodontally healthy and diseased patients. J Clin Periodontol. 41, 1027–1036 (2014). 25139407 10.1111/jcpe.12302 PMC4213353

[b31] US Government Printing Office. Series 1,1. DHHS publication (PHS) 94 1308. ed. (National Center for Health Statistics) (Washington, DC, 1994).

[b32] Sakellari D., Socransky S. S., Dibart S., Eftimiadi C. & Taubman M. A. Estimation of serum antibody to subgingival species using checkerboard immunoblotting. Oral Microbiol Immunol. 12, 303–310 (1997). 9467384 10.1111/j.1399-302x.1997.tb00395.x

[b33] Dye B. A. . Serum antibodies to periodontal bacteria as diagnostic markers of periodontitis. J Periodontol. 80, 634–647 (2009). 19335084 10.1902/jop.2009.080474

[b34] Gunter E. W., Lewis B. G. & Koncikowski S. M. ed (National Center for Environmental Health Centers for Disease Control and Prevention, and National Center for Health Statistics.) (Atlanta, GA., 1996).

[b35] Gunter E. W. & McQuillan G. Quality control in planning and operating the laboratory component for the Third National Health and Nutrition Examination Survey. J Nutr. 120, 1451–1454 (1990). 2243286 10.1093/jn/120.suppl_11.1451

[b36] Drury T. F. . An overview of the oral health component of the 1988–1991 national health and nutrition examination survey (NHANES III-Phase 1). J Dent Res 75, 620–630 (1996). 8594086 10.1177/002203459607502S02

[b37] Pussinen P. J. . Periodontal pathogen carriage, rather than periodontitis, determines the serum antibody levels. J Clin Periodontol. 38, 405–411 (2011). 21362013 10.1111/j.1600-051X.2011.01703.x

[b38] Suzuki N., Yoneda M. & Hirofuji T. Mixed red-complex bacterial infection in periodontitis. Int J Dent. 2013, 587279 (2013). 23533413 10.1155/2013/587279 PMC3606728

[b39] Hajishengallis G. . Low-abundance biofilm species orchestrates inflammatory periodontal disease through the commensal microbiota and complement. Cell Host Microbe. 10, 497–506 (2011). 22036469 10.1016/j.chom.2011.10.006 PMC3221781

[b40] Taylor G. W. Bidirectional interrelationships between diabetes and periodontal diseases: an epidemiologic perspective. Ann Periodontol. 6, 99–112 (2001). 11887478 10.1902/annals.2001.6.1.99

[b41] Slocum C., Kramer C. & Genco C. A. Immune dysregulation mediated by the oral microbiome: potential link to chronic inflammation and atherosclerosis. J Intern Med. 280, 114–128 (2016). 26791914 10.1111/joim.12476

[b42] Zeng X. T. . Periodontal Disease and Incident Lung Cancer Risk: A Meta-Analysis of Cohort Studies. J Periodontol. 87, 1158–1164 (2016). 27294431 10.1902/jop.2016.150597

[b43] Whitmore S. E. & Lamont R. J. Oral bacteria and cancer. PLoS Pathog. 10, e1003933 (2014). 24676390 10.1371/journal.ppat.1003933 PMC3968118

[b44] Hajishengallis G. Periodontitis: from microbial immune subversion to systemic inflammation. Nat Rev Immunol. 15, 30–44 (2015). 25534621 10.1038/nri3785 PMC4276050

[b45] Slocum C. . Distinct lipid a moieties contribute to pathogen-induced site-specific vascular inflammation. PLoS Pathog. 10, e1004215 (2014). 25010102 10.1371/journal.ppat.1004215 PMC4092147

[b46] Bullon P., Newman H. N. & Battino M. Obesity, diabetes mellitus, atherosclerosis and chronic periodontitis: a shared pathology via oxidative stress and mitochondrial dysfunction? Periodontol 2000 64, 139–153 (2014). 24320961 10.1111/j.1600-0757.2012.00455.x

[b47] Varela-López A., Quiles J.L., Cordero M., Giampieri F. & Bullón P. Oxidative Stress and Dietary Fat Type in Relation to Periodontal Disease. Antioxidants (Basel). 4, 322–344 (2015). 26783708 10.3390/antiox4020322 PMC4665476

[b48] Katz J. . Expression of the receptor of advanced glycation end products in gingival tissues of type 2 diabetes patients with chronic periodontal disease: a study utilizing immunohistochemistry and RT-PCR. J Clin Periodontol. 32, 40–44 (2005). 15642057 10.1111/j.1600-051X.2004.00623.x

[b49] Wang Z. . Gut flora metabolism of phosphatidylcholine promotes cardiovascular disease. Nature. 472, 57–63 (2011). 21475195 10.1038/nature09922 PMC3086762

[b50] Arimatsu K. . Oral pathobiont induces systemic inflammation and metabolic changes associated with alteration of gut microbiota. Sci Rep. 4, 4828 (2014). 24797416 10.1038/srep04828 PMC4010932

[b51] Chapple I. L. . Adjunctive daily supplementation with encapsulated fruit, vegetable and berry juice powder concentrates and clinical periodontal outcomes: a double-blind RCT. J Clin Periodontol. 39, 62–72 (2012). 22093005 10.1111/j.1600-051X.2011.01793.x PMC3267052

[b52] Loozen G. . Inter-bacterial correlations in subgingival biofilms: a large-scale survey. J Clin Periodontol 41, 1–10 (2014). 24102517 10.1111/jcpe.12167

[b53] Sarkar J., McHardy I. H., Simanian E. J., Shi W. & Lux R. Transcriptional responses of Treponema denticola to other oral bacterial species. PLOS One 9, e88361 (2014). 24505483 10.1371/journal.pone.0088361 PMC3914990

[b54] Noble J. M. . Periodontitis is associated with cognitive impairment among older adults: analysis of NHANES-III. J Neurol Neurosurg Psychiatry. 80, 1206–1211 (2009). 19419981 10.1136/jnnp.2009.174029 PMC3073380

