# Supplementary material for: Associations between Periodontal Microbiota and Death Rates
Source: Sci Rep. 2016 Oct 17;6:35428. doi: 10.1038/srep35428 (PMC5066247; doi:10.1038/srep35428)
Supplement: Supplementary Information [file srep35428-s1.pdf]

Associations between Periodontal Microbiota and Death Rates

Chung-Jung Chiu DDS PhD,[1]* Min-Lee Chang MS,[1] and Allen Taylor PhD[1]

[1]*Jean Mayer United States Department of Agriculture Human Nutrition Research Center on Aging, Tufts University, Boston, MA*.

*Correspondence to: Chung-Jung Chiu, Jean Mayer USDA Human Nutrition Research Center on Aging at Tufts University, 711 Washington Street, Boston, MA 02111, PHONE: 617-556-3157, FAX: 617-556-3132, E-mail: CJ.Chiu@tufts.edu

KEY WORDS: bacteria, periodontitis, mortality, diabetes, hypertension, National Health and Nutrition Examination Survey (NHANES), personalized medicine.

SUPPLEMENTARY FIGURE LEGENDS

Supplementary Figure 1. Cox proportional hazard regression analysis relating 21 anti-periodontal microbes IgGs to all-cause (a), diabetes-related (b), and hypertension-related (c) death rates. Models were adjusted for age, sex, race, education level, smoking status, body mass index, drinking alcohol (at least 12 drinks in the past 12 months), serum levels of C reactive protein, and the sampling weights in the Third National Health and Nutrition Examination Survey.

Natural log-transformed IgG variables were used in the analysis. In all-cause mortality analysis, significant (P<0.05) or marginally significant (0.05<P<0.1) HRs (P values) included 0.891 (0.031) for PI, 1.169 (0.029) for AAMX, 1.132 (0.001) for EC, 0.926 (0.056) for CO, 0.916 (0.007) for AN, and 0.874 (0.011) for SM. In diabetes-related mortality analysis, they were 1.379 (0.063) for PN, 1.997 (0.031) for AAMX, 0.451 (0.002) for AAY4, 0.771 (0.095) for AN, and 0.728 (0.097) for SN. In hypertension-related mortality analysis, only AN showed a marginally significant association (HR=0.867; P= 0.072).

IgG: immunoglobulin G.

PLS: partial least squares.

HR: hazard ratio.

CI: confidence interval.

PGMX: *Porphyromonas gingivalis*, a mixed suspension of ATCC strains #33277 and #53978.

PI: *Prevotella intermedia* ATCC#25611.

PN: *Prevotella nigrescens* ATCC#33563.

TF: *Tannerella forsythia* ATCC#43037.

AAMX: *Aggregatibacter actinomycetemcomitans*, a mixed suspension of three strains (ATCC#43718, #29523 and #33384).

AA29: *Aggregatibacter actinomycetemcomitans* serotype a (ATCC strain #29523).

AAY4: *Aggregatibacter actinomycetemcomitans* serotype b (ATCC strain #43718).

FN: *Fusobacterium nucleatum* ATCC#10953.

SO: *Streptococcus oralis* ATCC#35037.

MM: *Micromonas micros* ATCC #33270 .

CR: *Campylobacter rectus* ATCC#33238.

EC: *Eikenella corrodens* ATCC#23834.

EN: *Eubacterium nodatum* ATCC#33099.

SI: *Streptococcus intermedius* ATCC#27335.

CO: *Capnocytophaga ochracea* ATCC#33624.

VP: *Veillonella parvula* ATCC#10790.

AN: *Actinomyces naeslundii* ATCC#49340.

PM: *Prevotella melaninogenica* ATCC#25845.

SN: *Selenomonas noxia* ATCC#43541.

TD: *Treponema denticola* OMGS#3271.

SM: *Streptococcus mutans* ATCC#25175.

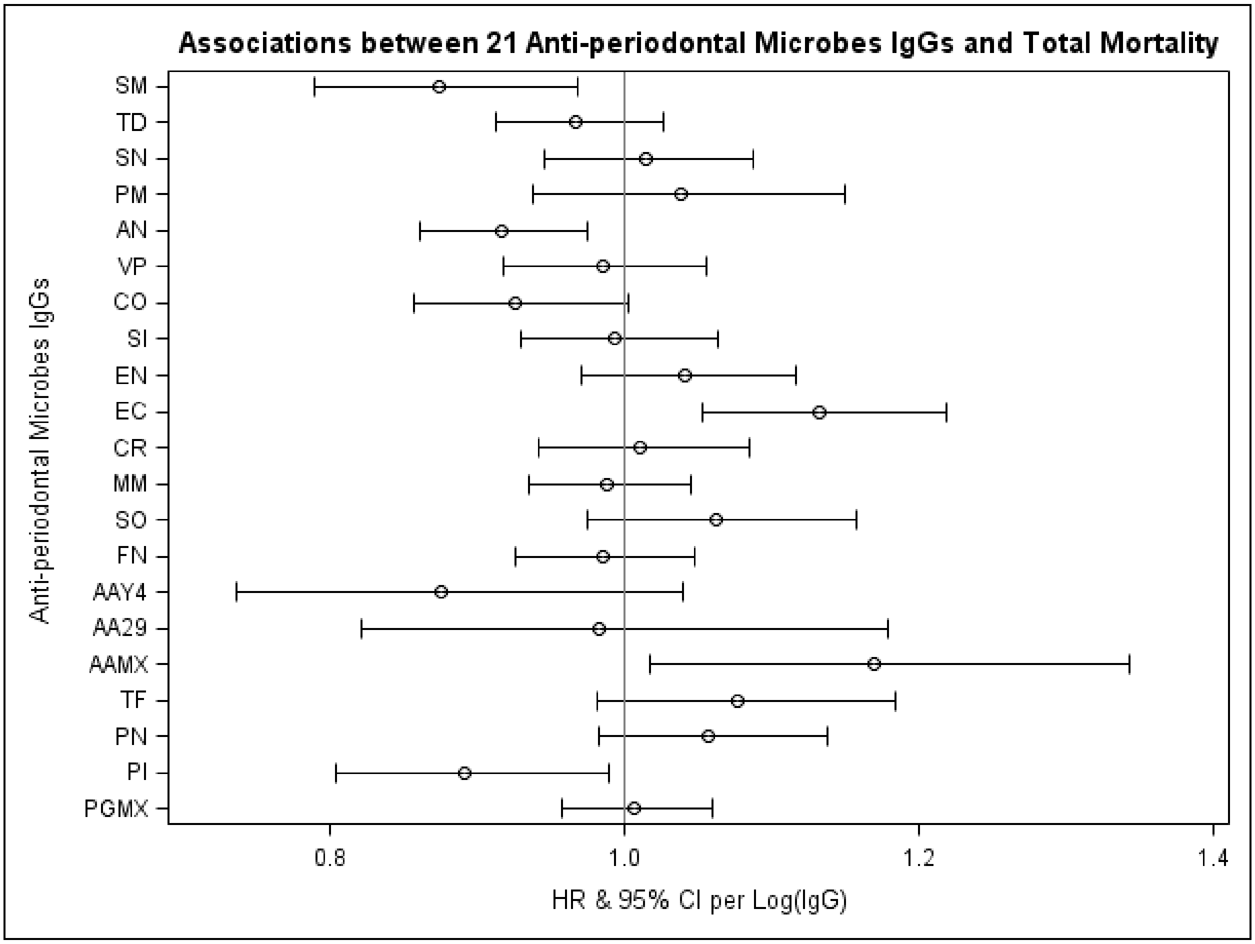

Associations between 21 Anti-periodontal Microbes IgGs and Total Mortality
Anti-periodontal Microbes IgGs
SM
TD
SN
PM
AN
VP
CO
SI
EN
EC
CR
MM
SO
FN
AAY4
AA29
AAMX
TF
PN
PI
PGMX
0.8
1.0
1.2
1.4
HR & 95% CI per Log(IgG)

(a)

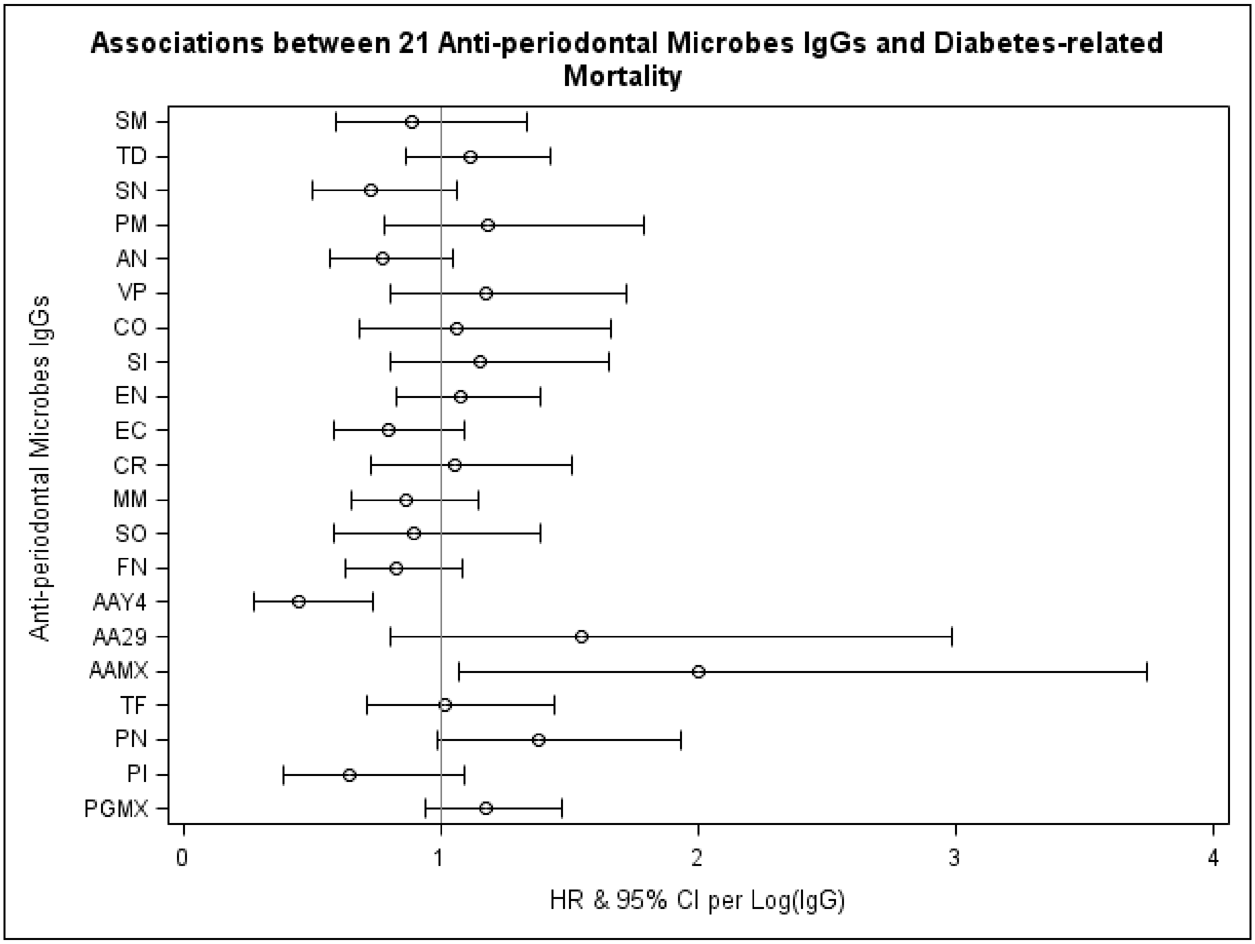
Associations between 21 Anti-periodontal Microbes IgGs and Diabetes-related Mortality
Anti-periodontal Microbes IgGs
SM
TD
SN
PM
AN
VP
CO
SI
EN
EC
CR
MM
SO
FN
AAY4
AA29
AAMX
TF
PN
PI
PGMX
0
1
2
3
4
HR & 95% CI per Log(IgG)

(b)

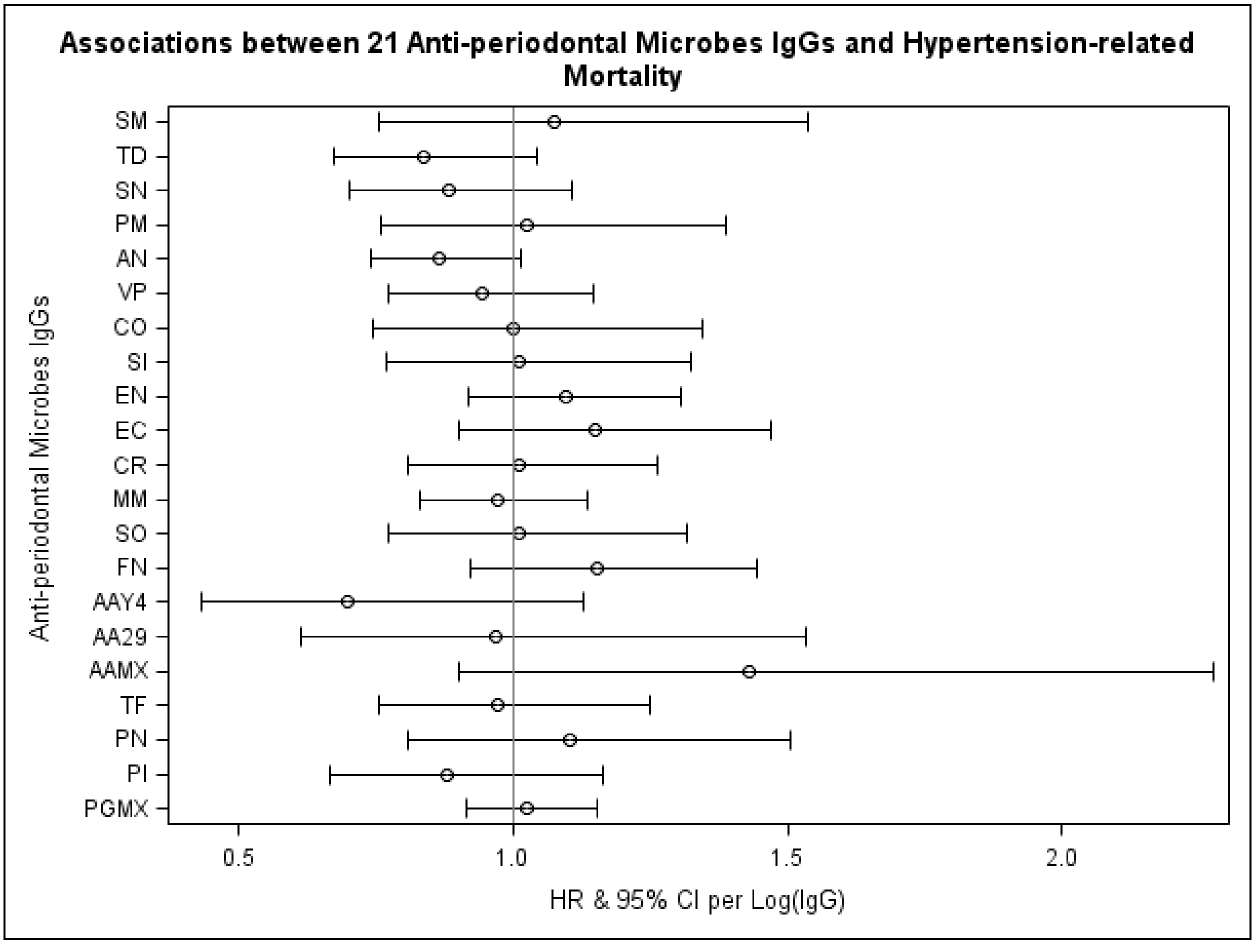

Associations between 21 Anti-periodontal Microbes IgGs and Hypertension-related Mortality
SM
TD
SN
PM
AN
VP
CO
SI
EN
EC
CR
MM
SO
FN
AAY4
AA29
AAMX
TF
PN
PI
PGMX
Anti-periodontal Microbes IgGs
0.5
1.0
1.5
2.0
HR & 95% CI per Log(IgG)

(c)
